# Acompanhamento de Dois Anos de Pacientes com Cardiopatia Isquêmica Crônica em um Centro Especializado no Brasil

**DOI:** 10.36660/abc.20220440

**Published:** 2023-10-16

**Authors:** Eduardo Martelli Moreira, Henrique Trombini Pinesi, Eduardo Bello Martins, Fábio Grunspun Pitta, Paula Mathias Paulino Bolta, Carlos Alexandre Wainrober Segre, Desiderio Favarato, Fabiana Hanna Rached, Whady Armindo Hueb, Eduardo Gomes Lima, Roberto Kalil, Cibele Larrosa Garzillo, Carlos Vicente Serrano

**Affiliations:** 1 Hospital das Clínicas Faculdade de Medicina Universidade de São Paulo São Paulo SP Brasil Instituto do Coração do Hospital das Clínicas da Faculdade de Medicina da Universidade de São Paulo, São Paulo, SP – Brasil

**Keywords:** Isquemia Miocárdica, LDL-Colesterol, Infarto do Miocárdio, Qualidade da Assistência à Saúde, Angina Pectoris

## Abstract

**Fundamento:**

A incidência de eventos cardiovasculares em pacientes com doença cardíaca isquêmica crônica (DCIC) pode variar significativamente entre os países. Embora populoso, o Brasil é frequentemente sub-representado nos registros internacionais.

**Objetivos:**

Este estudo teve como objetivo descrever a qualidade do atendimento e a incidência de eventos cardiovasculares em dois anos, além de fatores prognósticos associados em pacientes com DCIC em um centro terciário de saúde pública no Brasil.

**Métodos:**

Pacientes com DCIC que compareceram para avaliação clínica no Instituto do Coração (São Paulo, Brasil) foram cadastrados e acompanhados por dois anos. O desfecho primário foi um composto de infarto do miocárdio (IM), acidente vascular encefálico ou morte. Um nível de significância de 0,05 foi adotado.

**Resultados:**

De janeiro de 2016 a dezembro de 2018, 625 participantes foram incluídos no estudo. As características basais mostram que 33,1% eram mulheres, a idade mediana era de 66,1 [59,6 – 71,9], 48,6% tinham diabetes, 83,1% tinham hipertensão, 62,6% tinham IM prévio e 70,4% passaram por algum procedimento de revascularização. Em um acompanhamento mediano de 881 dias, 37 (7,05%) desfechos primários foram observados. Após ajustes, idade, acidente vascular encefálico prévio e colesterol LDL foram independentemente associados ao desfecho primário. Comparando a linha de base com o acompanhamento, os participantes relataram alívio da angina com base na escala da Sociedade Cardiovascular Canadense (SCC) de acordo com as seguintes porcentagens: 65,7% vs. 81,7% eram assintomáticos e 4,2% vs. 2,9% eram SCC 3 ou 4 (p < 0,001). Eles também relataram melhor qualidade na prescrição de medicamentos: 65,8% vs. 73,6% (p < 0,001). No entanto, não houve melhora no colesterol LDL ou no controle da pressão arterial.

**Conclusão:**

O presente estudo mostra que pacientes com DCIC apresentaram uma incidência de 7,05% do desfecho primário composto em um período de dois anos, sendo a diminuição do colesterol LDL o único fator de risco modificável associado ao prognóstico.

## Figura Central

**Figure:**
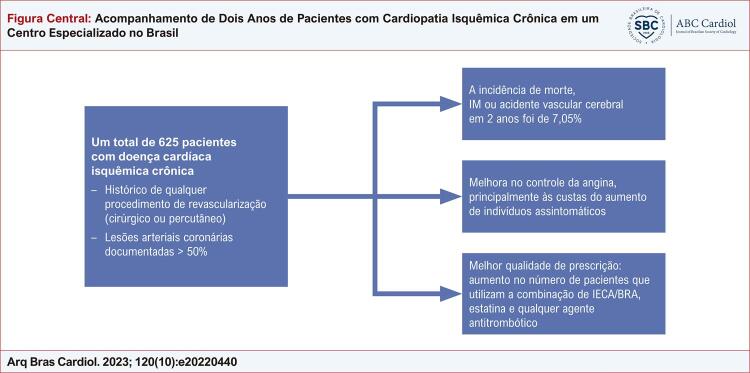
: Acompanhamento de Dois Anos de Pacientes com Cardiopatia Isquêmica Crônica em um Centro Especializado no Brasil

Desenho e principais conclusões do artigo. IM: infarto do miocárdio; inibidor da enzima conversora de angiotensina; BRA: bloqueadores dos receptores da angiotensina.

## Introdução

Das 55,9 milhões de mortes registradas globalmente em 2017, 17,8 milhões foram atribuídas a doenças cardiovasculares, predominantemente doenças coronarianas e cerebrovasculares.^1^ No Brasil, essas enfermidades respondem por cerca de um terço do total de mortes.^2^ Apesar de as taxas de mortalidade por doenças cardíacas isquêmicas crônicas (DCIC) padronizadas para o Brasil serem comparáveis às dos Estados Unidos e do Reino Unido, as marcantes discrepâncias em relação aos sistemas de saúde pública, Produto Interno Bruto, prevalência de fatores de risco e outras particularidades regionais podem apresentar desafios únicos para o manejo adequado da doença.^3^

O Brasil tem participado de registros internacionais de DCIC, embora com menos pacientes do que seria esperado devido ao tamanho de sua população.^4,5^ Consequentemente, a caracterização adequada de pacientes brasileiros com DCIC é insatisfatória. As sociedades cardiovasculares brasileiras e outros pesquisadores também reconheceram essa necessidade não atendida e já publicaram alguns de seus dados, porém ainda subsistem aspectos essenciais pendentes,^6-9^ que incluem os resultados de médio e longo prazo desses pacientes. Por exemplo, o maior registro brasileiro de pacientes com DCIC, o Registro de Prática Clínica em Pacientes com Alto Risco Cardiovascular (REACT), publicou apenas recentemente seus resultados de um ano, relatando uma incidência de morte de 4,92%.^6^

Estudos epidemiológicos na população brasileira indicaram baixas taxas de alcance dos objetivos terapêuticos adequados e de utilização de medicamentos que influenciam o prognóstico.^2,6^ Contudo, tais estudos foram conduzidos com a população em geral ou pacientes de alto risco cardiovascular, não se focando especificamente em indivíduos com doença arterial coronariana.

Portanto, buscamos melhorar a caracterização dos pacientes com DCIC no Brasil. O objetivo deste estudo é apresentar os números de ocorrências de óbito, infarto do miocárdio (IM) ou acidente vascular encefálico ao longo de dois anos em pacientes brasileiros com DCIC que foram acompanhados em um centro de saúde pública de nível terciário. Como objetivo secundário, tentamos identificar fatores determinantes para o prognóstico e avaliar o uso de medicamentos e o controle dos fatores de risco nesses pacientes.

## Métodos

Trata-se de um relato de acompanhamento de dois anos sobre um registro observacional prospectivo. O registro está atualmente em uso e este relatório refere-se a esse período específico. Procuramos estar em conformidade com as diretrizes STROBE.^10^

### Ambiente e pacientes

O estudo foi realizado no Instituto do Coração (InCor), em São Paulo, Brasil. O InCor é um centro terciário de referência para pacientes cardiopatas de alta complexidade e alto risco. Esses pacientes possuem plano de saúde público e provêm principalmente do estado de São Paulo, bem como de outras partes do país. De janeiro de 2016 a dezembro de 2018, registramos pacientes com DCIC estável que realizaram tratamento e atendimento em nosso ambulatório. Para serem elegíveis, os pacientes deveriam ter histórico de cirurgia de revascularização do miocárdio, intervenção coronária percutânea ou lesões documentadas da artéria coronária > 50%. Nesta análise, não incluímos pacientes com síndromes coronarianas agudas. No entanto, esses pacientes eram elegíveis para inclusão após a alta, caso fossem acompanhados no ambulatório (Figura Central).

Nesta investigação, o diagnóstico de diabetes foi baseado em pacientes com hemoglobina glicada igual ou superior a 6,5% ou em uso de medicação antidiabética. Hipertensão foi definida como uso de qualquer agente anti-hipertensivo.

Todos os pacientes forneceram consentimento assinado.

### Medições e resultados

A coleta de dados foi padronizada e os pacientes foram avaliados na linha de base e a cada ano depois disso. O acompanhamento foi feito pessoalmente sempre que possível ou remotamente por meio de ligações telefônicas.

O desfecho primário foi o composto de morte, IM ou acidente vascular encefálico. Não houve comissão julgadora do evento. Em vez disso, confiamos em registros de saúde e relatórios de pacientes. Como o InCor é referência no tratamento da DCIC, muitos desses pacientes foram atendidos em nossas instalações. Quando não, eram solicitados que trouxessem registros de saúde de outros prestadores. A morte foi verificada duas vezes em bancos de dados governamentais. Os desfechos secundários compreenderam a incidência de morte ou IM, além do composto de morte e IM. Todas as análises foram de natureza exploratória.

### Análise estatística

A normalidade foi avaliada pelo teste de Shapiro-Wilk. Os dados contínuos não apresentaram distribuição normal e, portanto, foram descritos por meio de mediana e intervalo interquartil (percentil 25 – 75). Os dados categóricos são apresentados como valores absolutos e porcentagens. Este é um registro contínuo de pacientes com DCIC, sem tamanho de estudo pré-especificado.

Os desfechos primários e secundários são relatados como incidências de Kaplan-Meier em dois anos e seus respectivos intervalos de confiança de 95%. Hazard ratios (HR) dos fatores prognósticos foram estimados de acordo com a modelagem de riscos proporcionais de Cox. A modelagem multivariada foi feita com um algoritmo de seleção regressiva de variáveis com *p-to-remove* de 0,05. Todos os fatores com valor de p < 0,10 nas análises univariadas foram incluídos no modelo multivariado inicial. Foram analisadas as alterações nos parâmetros clínicos e laboratoriais entre as avaliações utilizando os testes de McNemar e McNemar-Bowker para variáveis categóricas, além do teste de postos sinalizados de Wilcoxon para variáveis contínuas.

Todas as análises foram feitas no software R (versão 4.2.2).^11^ Um nível de significância de 0,05 foi adotado.

## Resultados

De janeiro de 2016 a dezembro de 2018, 625 participantes com DCIC conhecida foram incluídos no registro, e as características basais estão ilustradas na Tabela 1. No tempo mediano de 881 dias (IIQ: 613-1071), 553 pacientes foram reavaliados.

**Tabela 1:** – Características da linha de base

	Global (n=625)
Mulheres, n	207 (33,1%)
Idade, anos	66,1 [59,6 – 71,9]
IMC, kg/m^2^	27,3 [24,7 – 30,8]
IM prévio, n	391 (62,6%)
CRM prévia, n	202 (32,4%)
ICP prévia, n	296 (47,4%)
ICP ou CRM prévias, n	423 (67,7%)
Acidente vascular encefálico prévio, n	33 (5,3%)
DAP, n	34 (5,4%)
IM, acidente vascular encefálico ou procedimento de revascularização prévios, n	543 (86,9%)
Hipertensão, n	517 (83,1%)
Diabetes, n	304 (48,6%)
Insuficiência renal crônica, n*	205 (34,7%)
Fração de ejeção < 40%, n	110 (24,8%)

As variáveis contínuas são descritas como medianas [intervalo interquartil]. Insuficiência renal crônica: depuração de creatinina pelo método de Cockroft-Gault < 60 ml/min. IMC: índice de massa corporal; IM: infarto do miocárdio; CRM: cirurgia de revascularização miocárdica; ICP: intervenção coronária percutânea; DAP: doença arterial periférica.

A Tabela 2 compara o uso de medicamentos na linha de base e durante o acompanhamento. Não houve diferença significativa na prescrição global de agentes antitrombóticos (96,6% para 95,3%, p = 0,32). O uso de estatinas diminuiu (96,6% para 93,7%, p = 0,02). Por outro lado, a prescrição de inibidores da enzima conversora de angiotensina (IECA) ou bloqueadores dos receptores de angiotensina (BRA) aumentou (69,4% para 79,7%, p < 0,001). O uso de metformina e sulfonilureias também aumentou (36,7% para 43,9%, p < 0,001; 18,3% para 22,4%, p = 0,01, respectivamente). Não houve diferença na prescrição de insulina (11,2% para 13,4%, p = 0,11) ou outros antidiabéticos (2,7% para 2,7%, p = 1,00). Considerando a prescrição combinada de medicamentos com benefício cardiovascular conhecido para a população com DCIC (IECA ou BRA mais estatina, mais qualquer agente antitrombótico), uma proporção maior de pacientes estava em tratamento médico ideal no acompanhamento, em comparação com a linha de base: 65,8% vs. 73,6%, p < 0,001.

**Tabela 2:** – Medicamentos na linha de base e no acompanhamento

	Linha de base (n = 553)	Acompanhamento (n = 553)	Valor-p
AAS	516 (93,3%)	491 (88,8%)	< 0,001
Outro agente antiplaquetário	133 (24,1%)	62 (11,2%)	< 0,001
Anticoagulantes orais	28 (5,1%)	34 (6,1%)	0,21
Qualquer agente antitrombótico	534 (96,6%)	527 (95,3%)	0,32
Estatina	534 (96,6%)	518 (93,7%)	0,02
Ezetimiba	15 (2,7%)	10 (1,8%)	0,33
Fibratos	31 (5,6%)	25 (4,5%)	0,42
IECA ou BRA	384 (64,9%)	441 (79,7%)	< 0,001
Betabloqueador	492 (89,0%)	468 (84,6%)	< 0,001
Bloqueador de canais de cálcio	208 (37,6%)	227 (41,0%)	0,09
Nitratos	163 (29,5%)	116 (21,0%)	< 0,001
Ivabradina	2 (0,4%)	3 (0,5%)	1,0
Trimetazidina	5 (0,9%)	6 (1,1%)	1,0
Metformina	203 (36,7%)	243 (43,9%)	< 0,001
Sulfonilureias	101 (18,3%)	124 (22,4%)	0,01
Insulina	62 (11,2%)	74 (13,4%)	0,11
Outros agentes antidiabéticos	15 (2,7%)	15 (2,7%)	1,00
IECA ou BRA + estatina + qualquer agente antitrombótico	364 (65,8%)	407 (73,6%)	< 0,001

AAS: ácido acetilsalicílico; IECA: inibidor da enzima conversora de angiotensina; BRA: bloqueadores dos receptores da angiotensina.

No acompanhamento, os pacientes estavam menos sintomáticos (p < 0,001). A pressão arterial sistólica e diastólica, a frequência cardíaca, o colesterol LDL e o colesterol HDL foram significativamente diferentes das medidas basais (p < 0,05 para todas as análises), embora apenas levemente em intensidade (Tabela 3). Colesterol total, triglicerídeos e glicose não foram significativamente diferentes das medições iniciais.

**Tabela 3:** – Achados clínicos na linha de base e no acompanhamento

	Linha de base (n = 553)	Acompanhamento (n = 553)	Alteração mediana [25º - 75º percentil]	Valor-p
**Intensidade da angina, n**				< 0,001
Sem angina	358 (66%)	445 (82%)		
SCC 1	71 (13%)	39 (7%)		
SCC 2	93 (17%)	45 (8%)		
SCC 3 ou 4	23 (4%)	16 (3%)		
Pressão arterial sistólica, mmHg	130 [120 – 140]	130 [120 – 150]	0 [-10 – 20]	< 0,01
Pressão arterial diastólica, mmHg	80 [70 – 80]	80 [70 – 90]	0 [-10 – 10]	0,03
Frequência cardíaca, bpm	64 [60 – 72]	68 [61 – 72]	0 [-5 – 8]	0,02
Colesterol sérico, mg/dL	152 [130 – 184]	155 [131 – 188]	1 [-16 – 22]	0,054
Colesterol LDL, mg/dL	83 [64 – 108]	83,5 [64 – 112]	1,5 [-13 – 18]	0,03
Colesterol HDL, mg/dL	43 [36 – 51]	41 [36 – 49,2]	-1 [-5 – 3]	0,01
Triglicerídeos, mg/dL	119 [82 – 159]	118 [85 – 168]	-1 [-26,5 – 32,5]	0,64
Glicose, mg/dL	115 [103 – 143]	115 [103 – 154]	0 [-11 – 13,5]	0,32

As variáveis contínuas são descritas como medianas [intervalo interquartil]. SCC: Sociedade Cardiovascular Canadense; Colesterol LDL: colesterol de lipoproteína de baixa densidade; Colesterol HDL: colesterol de lipoproteína de alta densidade.

**Figura 1:**
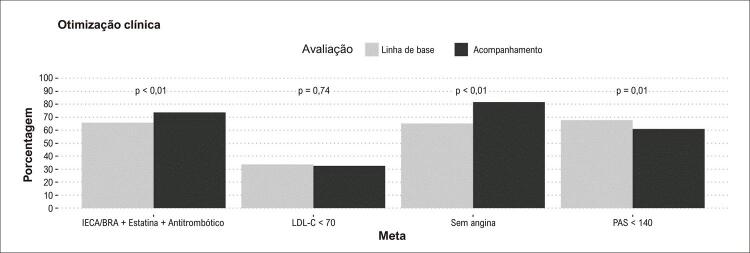
– Número de pacientes com objetivos clínicos alcançados. IECA: inibidor da enzima conversora de angiotensina; BRA: bloqueadores dos receptores da angiotensina; Antitrombóticos: antiplaquetários e/ou anticoagulantes orais; LDL-c: colesterol lipoproteico de baixa densidade; PAS: pressão arterial sistólica.

**Figura 2:**
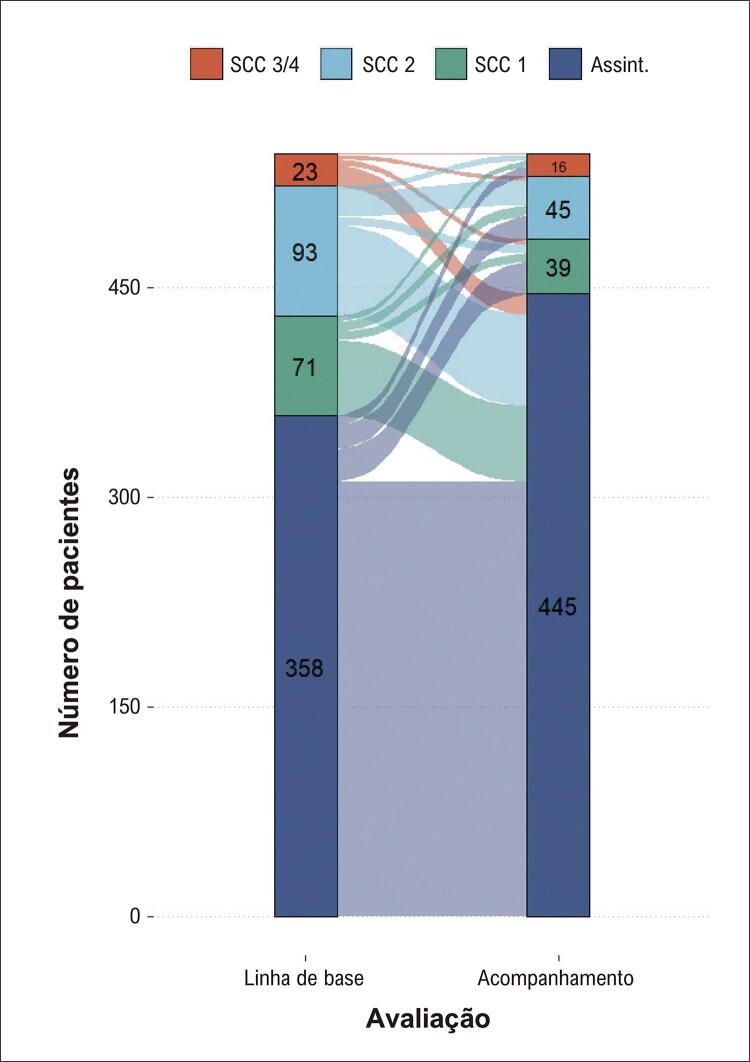
– Intensidade da angina na linha de base e no acompanhamento. SCC: Sociedade Cardiovascular Canadense; Assint.: assintomáticos.

Em comparação com a avaliação inicial, mais pacientes estavam assintomáticos (definidos como SCC 0; 65,2% vs 81,7%, p < 0,01) e menos pacientes atingiram a meta terapêutica de PAS < 140 mmHg (67,8% vs 61,0%, p = 0,01) no acompanhamento (Figura 1). Aproximadamente um terço dos pacientes atingiu a meta terapêutica de LDL-c < 70 mg/dL, tanto no início quanto no acompanhamento (p = 0,74). Dos 358 pacientes assintomáticos na linha de base, 47 (13,1%) desenvolveram angina durante o acompanhamento (Figura 2). Por outro lado, 54/71 (76,1%), 65/93 (69,9%) e 15/23 (65,2%) daqueles com angina graus 1, 2 ou 3/4, respectivamente, tornaram-se assintomáticos.

**Figura 3:**
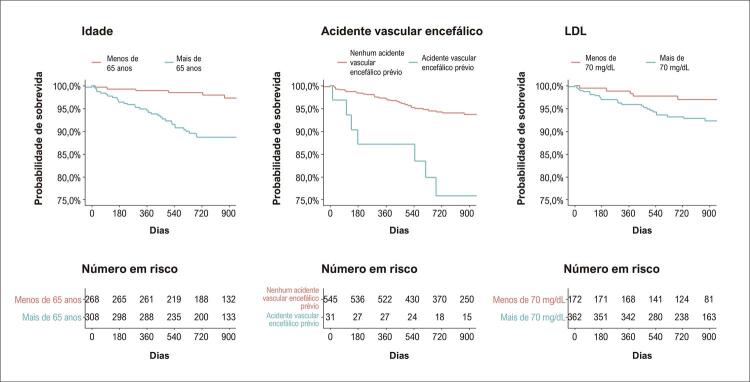
– Incidência de infarto do miocárdio, acidente vascular encefálico ou morte, estratificada por fatores prognósticos independentes. LDL: lipoproteína de baixa densidade.

Após um acompanhamento mediano de 881 dias, 37 eventos foram registrados para o desfecho primário (Tabela 4). A incidência de morte, IM ou acidente vascular encefálico em dois anos foi de 7,05%. Na análise não ajustada (Tabela 5), idade (HR de 1,58 por cinco anos, IC de 95% 1,35 - 1,85), acidente vascular encefálico anterior (HR de 3,11, IC de 95% 1,38 - 7,00), colesterol LDL (HR de 1,20 por aumento de 10 mg/dL, IC de 95% 1,11 - 1,30) e colesterol total (HR de 1,14 por 10 mg/dL, IC de 95% 1,07 - 1,22) foram associados a um aumento no desfecho primário. Após ajustes, idade (HR de 1,61 por aumento de cinco anos, IC de 95% 1,32 - 1,97), acidente vascular encefálico prévio (HR de 3,65, IC de 95% 1,48 - 9,00) e colesterol LDL (HR de 1,23 por 10 mg/dL, IC de 95% 1,14 - 1,33) foram independentemente associados ao desfecho primário (Figura 3).

**Tabela 4:** – Incidência de desfechos em dois anos

	Número de eventos	Incidência em 2 anos (IC de 95%)
Morte, IM, ou acidente vascular encefálico	37	7,05% (4,81 - 9,23)
Morte ou IM	35	6,67% (4,49 - 8,80)
Óbito	31	5,87% (3,83 - 7,87)

IM: infarto do miocárdio; IC: intervalo de confiança.

**Tabela 5:** – Modelos de riscos proporcionais de Cox ajustados e não ajustados para incidência de infarto do miocárdio, acidente vascular encefálico ou morte

	Não ajustado		Ajustado*	
	Hazard Ratio (IC de 95%)	Valor-p	Hazard Ratio (IC de 95%)	Valor-p
Mulheres	0,97 (0,52; 1,80)	0,93		
Idade, por 5 anos	1,58 (1,35; 1,85)	< 0,005	1,61 (1,32; 1,97)	< 0,001
IMC, 5 kg/m^2^	0,83 (0,60; 1,16)	0,28		
IM prévio	1,40 (0,74; 2,67)	0,30		
CRM prévia	1,79 (1,00; 3,20)	0,05		
ICP prévia	0,72 (0,40; 1,30)	0,28		
Acidente vascular encefálico prévio	3,11 (1,38; 7,00)	0,01	3,65 (1,48; 9,00)	< 0,001
DAP	0,30 (0,04; 2,17)	0,23		
Diabetes	0,88 (0,49; 1,58)	0,67		
Fração de ejeção, por 5%	0,90 (0,79; 1,02)	0,10		
Angina	1,04 (0,57; 1,89)	0,90		
Hipertensão	1,50 (0,59; 3,81)	0,39		
FC, por 10 bpm	0,79 (0,55; 1,15)	0,22		
LDL-c, 10 mg/dL	1,20 (1,11; 1,30)	< 0,001	1,23 (1,14; 1,33)	< 0,001
HDL-c, 10 mg/dL	1,06 (0,80; 1,41)	0,69		
Colesterol, por 10 mg/dL	1,14 (1,07; 1,22)	< 0,001		
Triglicerídeos, por 10 mg/dL	0,98 (0,94; 1,03)	0,41		

Os ajustes foram feitos de acordo com o algoritmo de seleção regressiva de variáveis, com p-to-enter < 0,10 e p-to-remove 0,05, conforme resultados univariados. IM: infarto do miocárdio CRM: cirurgia de revascularização do miocárdio; ICP: intervenção coronária percutânea; DAP: doença arterial periférica; FC: frequência cardíaca; IMC: índice de massa corporal; LDL-c: colesterol lipoproteico de baixa densidade; HDL-c: colesterol lipoproteico de alta densidade.

## Discussão

Até onde sabemos, este é um dos maiores registros de DCIC realizados no Brasil envolvendo pacientes estáveis. No geral, encontramos uma incidência de morte, IM ou acidente vascular encefálico em dois anos de 7,05%. Idade, acidente vascular encefálico prévio e colesterol LDL elevado foram os principais fatores de risco associados.

Apesar dos esforços que possam sugerir o contrário, os registros internacionais podem representar sobre ou sub-representação de países e regiões.^4,12^ Portanto, relatórios como este são valiosos para compreender melhor o fardo da DCIC nos níveis regional e mundial. Neste estudo, observamos que, apesar de um percentual elevado de prescrições de estatinas, menos de um terço dos pacientes apresentavam colesterol LDL inferior a 70 mg/dL e menos de 10% apresentavam níveis inferiores a 50 mg/dL.

O Estudo Longitudinal da Saúde do Adulto (ELSA), um registro brasileiro de alcance multirregional da população em geral, relatou que 9,4% dos participantes com alto risco de doença coronariana apresentaram níveis de colesterol LDL abaixo de 70 mg/dL.^8^ Outro registro REACT, também multicêntrico e brasileiro, que englobou pacientes com doença aterosclerótica manifesta e de alto risco, revelou que mais de 90% dos participantes em prevenção secundária apresentavam valores de colesterol LDL superiores a 50 mg/dL.^6^ Esses dados evidenciam que a maioria dos pacientes não atinge a meta estabelecida pelas diretrizes, de um nível de colesterol LDL entre 50-55 mg/dL.^13-15^ Quando se observa o colesterol não-HDL, apenas uma parcela menor (16,7%) dos pacientes alcançou a meta nacional de 80 mg/dL proposta pelas diretrizes.^13^

Isto é um fato alarmante, pois nossos estudos, assim como estudos anteriores,^16,17^ mostraram que níveis mais elevados de colesterol LDL estão associados a eventos cardiovasculares. Estudos anteriores estimaram que cada redução de 1 mmol/L (aproximadamente 38 mg/dL) no colesterol LDL leva a uma redução de 22% nos eventos cardiovasculares maiores ao longo de cinco anos.^16^ Como comparação, estimamos um aumento de 20% no risco para cada 10 mg/dL – uma estimativa de efeito mais alta. Além disso, identificamos que pouquíssimos pacientes receberam prescrição de ezetimiba (< 5%) e nenhum paciente estava em uso de inibidores da PCSK-9. A ezetimiba tem sido proposta como medicamento de segunda linha para pacientes que não atingiram níveis adequados de colesterol LDL.^13,15^ Atribuímos esse cenário ao fato de esses medicamentos não serem fornecidos pelo sistema público de saúde, usado pela maioria dos brasileiros (assim como nossos participantes). Acreditamos que os incluir no sistema público de saúde seria uma forma eficaz de melhorar a qualidade do atendimento aos pacientes com DCIC.

Em nosso estudo, o controle do LDL e do colesterol total durante o acompanhamento mostrou-se ruim (p < 0,05). Apesar disso, seu impacto foi pequeno do ponto de vista clínico (aumento de 4,1 mg/dL no LDL e aumento de 5 mg/dL no colesterol total). O mesmo pode ser observado para a pressão arterial sistólica (aumento de 3 mmHg no acompanhamento). Este declínio no controle das comorbidades ocorreu apesar da alta taxa de prescrição de estatinas (acima de 90% na linha de base e no acompanhamento) e do aumento na prescrição de inibidores da ECA ou BRA (p < 0,001). Houve também aumento na prescrição de antidiabéticos orais, que não melhoraram o controle glicêmico. Esses dados podem ser marcadores de má adesão ao tratamento.

Também pode acontecer que a diminuição do colesterol LDL seja um marcador de melhor adesão do paciente. Muitos estudos já demonstraram que a adesão do paciente representa um fator prognóstico significativo, chegando a ser considerada como “a próxima fronteira na melhoria da qualidade”.^18-20^ Relatos anteriores estimam que a falta de aderência está associada a um aumento de aproximadamente 18% nos eventos cardiovasculares graves.^20^ Embora não tenhamos avaliado isso de maneira formal, é possível interpretar a presença de níveis baixos de colesterol LDL como um indício da adesão ao uso de estatinas (e possivelmente do tratamento como um todo). De fato, pesquisas anteriores mostraram que até 30% dos brasileiros com doenças crônicas não transmissíveis podem ser não aderentes, e que os latino-americanos podem ser ainda mais não aderentes do que pacientes norte-americanos.^21,22^ Como a não adesão poderia estar relacionada com problemas do sistema de saúde pública e com os profissionais de saúde, investigações adicionais poderão ter um impacto sobre as políticas de saúde pública. Curiosamente, houve melhora no controle da angina. O número de pacientes sem angina aumentou (p < 0,001) e todas as outras classes funcionais tenderam a diminuir.

O Registro Prospectivo Observacional Longitudinal de Pacientes com Doença Arterial Coronariana Estável (CLARIFY, *ProspeCtive observational LongitudinAl RegIstry oF patients with stable coronary arterY disease*), um registro internacional contemporâneo de DCIC, relatou uma incidência de cinco anos de morte cardiovascular, IM ou acidente vascular encefálico de 9,5%.^23^ O Registro de Saúde Contínua para a Redução de Aterotrombose (REACH, *REduction of Atherothrombosis for Continued Health*), um registro internacional maior, mas um pouco mais antigo, relatou taxas de eventos de morte vascular, IM ou acidente vascular encefálico em um e três anos de 4,5% e 11,6%, respectivamente, para o subgrupo DCIC.^24^ O registro REACT relatou um taxa de mortalidade anual de 4,9%. Comparações diretas entre estudos são difíceis de interpretar devido a diferenças nos critérios de inclusão, população, período de tempo e fatores de risco analisados.

Ainda assim, nossos resultados parecem estar em linha com os registros internacionais e um pouco melhores que os registros brasileiros. Contudo, todos os estudos destacaram a variação substancial dos resultados de acordo com a localização geográfica, enfatizando ainda mais a relevância das pesquisas regionais e da inclusão adequada de diferentes regiões nos estudos internacionais, sendo fundamental para uma compreensão mais abrangente do impacto da aterosclerose globalmente.^6,23,24^

A doença polivascular demonstrou ser um importante fator de risco prognóstico, tanto em pacientes com síndrome coronariana crônica quanto aguda em todo o mundo.^12,25^ O mesmo ocorre com eventos isquêmicos prévios, com um aumento de risco associado de 40-50% para novos eventos cardiovasculares maiores.^6,12,23^ Coincidentemente, o AVE prévio foi um fator de risco independente em nosso registro. O AVE prévio em nossa população denota doença aterosclerótica polivascular (já que todos os pacientes tinham DCIC, de acordo com os critérios de inclusão). Na nossa população, o AVE prévio foi o fator de risco mais fortemente associado, representando um aumento estimado de quase quatro vezes na incidência do evento. No entanto, acidente vascular encefálico prévio e doença arterial periférica (dos membros inferiores) tiveram prevalência muito baixa na nossa coorte: 5,3% e 5,4%, respectivamente. Esses valores são inferiores aos relatados em outros estudos e podem indicar um grau de subdiagnóstico.^6,12,26^

A redução da fração de ejeção do ventrículo esquerdo, IM prévio e doença renal crônica são marcadores típicos de mau prognóstico em pacientes com DCIC.^23,27^ Não observamos essa correlação prognóstica em nosso estudo. Essa ausência de correlação poderia ser explicada, em parte, pelo número reduzido de pacientes com essas comorbidades. Outro fator que poderia contribuir para esse resultado é o acompanhamento mediano de dois anos e o baixo número de eventos em nossa população.

Este estudo apresentou diversas limitações. Primeiramente, incluímos apenas pacientes de um centro especializado, portanto não podemos pretender ser uma amostra representativa de DCIC no Brasil como um todo. Em segundo lugar, embora este seja um dos maiores registros de DCIC do país, ainda é relativamente pequeno em comparação com outros registros internacionais. O baixo número de eventos (n=37) também impede análises mais precisas de subgrupos ou fatores de risco. Em terceiro lugar, não tivemos um comitê formal de julgamento de eventos – contamos com relatórios de pacientes, registros de saúde e dados administrativos sempre que possível. Portanto, alguns eventos podem ter passado despercebidos. Em quarto lugar, todas as análises foram exploratórias e, portanto, há alta probabilidade de erro tipo I; sendo assim, deve-se ter cuidado ao interpretá-los.

## Conclusão

Em conclusão, mostramos que os pacientes com DCIC em nosso centro manifestaram uma taxa de 7,05% de mortalidade, acidente vascular encefálico ou IM ao longo de dois anos, alinhando-se aos principais registros internacionais. Ademais, identificamos o colesterol LDL como o principal fator de risco passível de modificação, apontando para a possibilidade de este ser um alvo valioso para avanços nos cuidados em saúde.

## References

[1] GBD 2017 Causes of Death Collaborators (2018). Global, Regional, and National Age-Sex-Specific Mortality for 282 Causes of Death in 195 Countries and Territories, 1980-2017: A Systematic Analysis for the Global Burden of Disease Study 2017. Lancet.

[2] Brant LCC, Nascimento BR, Passos VMA, Duncan BB, Bensenõr IJM, Malta DC (2017). Variations and Particularities in Cardiovascular Disease Mortality in Brazil and Brazilian States in 1990 and 2015: Estimates from the Global Burden of Disease. Rev Bras Epidemiol.

[3] Nowbar AN, Gitto M, Howard JP, Francis DP, Al-Lamee R (2019). Mortality from Ischemic Heart Disease. Circ Cardiovasc Qual Outcomes.

[4] Sorbets E, Greenlaw N, Ferrari R, Ford I, Fox KM, Tardif JC (2017). Rationale, Design, and Baseline Characteristics of the CLARIFY Registry of Outpatients with Stable Coronary Artery Disease. Clin Cardiol.

[5] Ohman EM, Bhatt DL, Steg PG, Goto S, Hirsch AT, Liau CS (2006). The REduction of Atherothrombosis for Continued Health (REACH) Registry: An International, Prospective, Observational Investigation in Subjects at Risk for Atherothrombotic Events-Study Design. Am Heart J.

[6] Silva PGMBE, Berwanger O, Precoma DB, Cavalcante MA, Vilela-Martin JF, Figueiredo EL (2021). Evaluation of 1-Year Follow-up of Patients Included in the Registry of Clinical Practice in Patients at High Cardiovascular Risk (REACT). Arq Bras Cardiol.

[7] Paez RP, Hossne NA, Santo JADE, Berwanger O, Santos RHN, Kalil RAK (2019). Coronary Artery Bypass Surgery in Brazil: Analysis of the National Reality Through the BYPASS Registry. Braz J Cardiovasc Surg.

[8] Lotufo PA, Santos RD, Figueiredo RM, Pereira AC, Mill JG, Alvim SM (2016). Prevalence, Awareness, Treatment, and Control of High Low-Density Lipoprotein Cholesterol in Brazil: Baseline of the Brazilian Longitudinal Study of Adult Health (ELSA-Brasil). J Clin Lipidol.

[9] Mattos LA (2011). Rationality and Methods of ACCEPT Registry - Brazilian Registry of Clinical Practice in Acute Coronary Syndromes of the Brazilian Society of Cardiology. Arq Bras Cardiol.

[10] von Elm E, Altman DG, Egger M, Pocock SJ, Gøtzsche PC, Vandenbroucke JP (2008). The Strengthening the Reporting of Observational Studies in Epidemiology (STROBE) Statement: Guidelines for Reporting Observational Studies. J Clin Epidemiol.

[11] The R Project for Statistical Computing (2020). R: A Language and Environment for Statistical Computing.

[12] Bhatt DL, Eagle KA, Ohman EM, Hirsch AT, Goto S, Mahoney EM (2010). Comparative Determinants of 4-year Cardiovascular Event Rates in Stable Outpatients at Risk of or with Atherothrombosis. JAMA.

[13] Précoma DB, Oliveira GMM, Simão AF, Dutra OP, Coelho OR, Izar MCO (2019). Updated Cardiovascular Prevention Guideline of the Brazilian Society of Cardiology - 2019. Arq Bras Cardiol.

[14] Knuuti J, Wijns W, Saraste A, Capodanno D, Barbato E, Funck-Brentano C (2020). 2019 ESC Guidelines for the Diagnosis and Management of Chronic Coronary Syndromes. Eur Heart J.

[15] Mach F, Baigent C, Catapano AL, Koskinas KC, Casula M, Badimon L (2020). 2019 ESC/EAS Guidelines for the Management of Dyslipidaemias: Lipid Modification to Reduce Cardiovascular Risk. Eur Heart J.

[16] Cholesterol Treatment Trialists’ CTT, Baigent C, Blackwell L, Emberson J, Holland LE, Reith C, Collaboration (2010). Efficacy and Safety of More Intensive Lowering of LDL Cholesterol: a Meta-Analysis of Data from 170,000 Participants in 26 Randomised Trials. Lancet.

[17] Vanassche T, Verhamme P, Anand SS, Shestakovska O, Fox KA, Bhatt DL (2020). Risk Factors and Clinical Outcomes in Chronic Coronary and Peripheral Artery Disease: An Analysis of the Randomized, Double-Blind COMPASS Trial. Eur J Prev Cardiol.

[18] Baroletti S, Dell’Orfano H (2010). Medication Adherence in Cardiovascular Disease. Circulation.

[19] Heidenreich PA (2004). Patient Adherence: The Next Frontier in Quality Improvement. Am J Med.

[20] Kumbhani DJ, Steg PG, Cannon CP, Eagle KA, Smith SC, Hoffman E (2013). Adherence to Secondary Prevention Medications and Four-Year Outcomes in Outpatients with Atherosclerosis. Am J Med.

[21] Tavares NU, Bertoldi AD, Mengue SS, Arrais PS, Luiza VL, Oliveira MA (2016). Factors Associated with Low Adherence to Medicine Treatment for Chronic Diseases in Brazil. Rev Saude Publica.

[22] Rodriguez F, Cannon CP, Steg PG, Kumbhani DJ, Goto S, Smith SC (2013). Predictors of Long-Term Adherence to Evidence-Based Cardiovascular Disease Medications in Outpatients with Stable Atherothrombotic Disease: Findings from the REACH Registry. Clin Cardiol.

[23] Sorbets E, Fox KM, Elbez Y, Danchin N, Dorian P, Ferrari R (2020). Long-Term Outcomes of Chronic Coronary Syndrome Worldwide: Insights from the International CLARIFY Registry. Eur Heart J.

[24] Alberts MJ, Bhatt DL, Mas JL, Ohman EM, Hirsch AT, Röther J (2009). Three-Year Follow-Up and Event Rates in the International REduction of Atherothrombosis for Continued Health Registry. Eur Heart J.

[25] Bhatt DL, Peterson ED, Harrington RA, Ou FS, Cannon CP, Gibson CM (2009). Prior Polyvascular Disease: Risk Factor for Adverse Ischaemic Outcomes in Acute Coronary Syndromes. Eur Heart J.

[26] Suárez C, Zeymer U, Limbourg T, Baumgartner I, Cacoub P, Poldermans D (2010). Influence of Polyvascular Disease on Cardiovascular Event Rates. Insights from the REACH Registry. Vasc Med.

[27] Sarnak MJ, Amann K, Bangalore S, Cavalcante JL, Charytan DM, Craig JC (2019). Chronic Kidney Disease and Coronary Artery Disease: JACC State-of-the-Art Review. J Am Coll Cardiol.

