# Supplementary material for: Acompanhamento de Dois Anos de Pacientes com Cardiopatia Isquêmica Crônica em um Centro Especializado no Brasil
Source: Arq Bras Cardiol. 2023 Oct 16;120(10):e20220440. [Article in Portuguese] doi: 10.36660/abc.20220440 (PMC10586816; doi:10.36660/abc.20220440)
Supplement: Translation

# Translation: Two-Year Follow-Up of Chronic Ischemic Heart Disease Patients in a Specialized Center in Brazil

**Authors:** Eduardo Martelli Moreira, Henrique Trombini Pinesi, Eduardo Bello Martins, Fábio Grunspun Pitta, Paula Mathias Paulino Bolta, Carlos Alexandre Wainrober Segre, Desiderio Favarato, Fabiana Hanna Rached, Whady Armindo Hueb, Eduardo Gomes Lima, Roberto Kalil, Cibele Larrosa Garzillo, Carlos Vicente Serrano

**Affiliations:** 1 Brazil Instituto do Coração do Hospital das Clínicas da Faculdade de Medicina da Universidade de São Paulo, São Paulo, SP – Brazil

**Keywords:** Myocardial Ischemia, Cholesterol, LDL, Myocardial Infarction, Quality of Health Care, Angina Pectoris

## Abstract

**Background:**

The incidence of cardiovascular events in patients with chronic ischemic heart disease (CIHD) may vary significantly among countries. Although populous, Brazil is often underrepresented in international records.

**Objectives:**

This study aimed to describe the quality of care and the two-year incidence of cardiovascular events and associated prognostic factors in CIHD patients in a tertiary public health care center in Brazil.

**Methods:**

Patients with CIHD who reported for clinical evaluation at Instituto do Coração (São Paulo, Brazil) were registered and followed for two years. The primary endpoint was a composite of myocardial infarction (MI), stroke, or death. A significance level of 0.05 was adopted.

**Results:**

From January 2016 to December 2018, 625 participants were included in the study. Baseline characteristics show that 33.1% were women, median age 66.1 [59.6 – 71.9], 48.6% had diabetes, 83.1% had hypertension, 62.6% had previous MI, and 70.4% went through some revascularization procedure. At a median follow-up (FU) of 881 days, we noted 37 (7.05%) primary endpoints. After adjustments, age, previous stroke, and LDL-cholesterol were independently associated with the primary endpoint. Comparing baseline versus FU, participants experienced relief of angina based on the Canadian Cardiovascular Society (CCS) scale according to the following percentages: 65.7% vs. 81.7% were asymptomatic and 4.2% vs. 2.9% CCS 3 or 4 (p < 0.001). They also experienced better quality of medication prescription: 65.8% vs. 73.6% (p < 0.001). However, there was no improvement in LDL-cholesterol or blood pressure control.

**Conclusion:**

This study shows that CIHD patients had a two-year incidence of the primary composite endpoint of 7.05%, and the reduction of LDL-cholesterol was the only modifiable risk factor associated with prognosis.

## Introduction

Out of the 55.9 million deaths worldwide in 2017, 17.8 million were due to cardiovascular diseases, mainly coronary artery and cerebrovascular diseases.^1^ In Brazil, they account for almost a third of the total deaths.^2^ Although Brazilian standardized death rates for chronic ischemic heart disease (CIHD) are similar to those in the US and the United Kingdom, the vast differences in public health care, gross domestic product, prevalence of risk factors, and other regional characteristics may provide unique challenges for the adequate management of the disease.^3^

Brazil has participated in international CIHD registries, albeit with fewer patients than it would be expected due to its population size.^4,5^ Consequently, adequate characterization of Brazilian CIHD patients is unsatisfactory. Brazilian cardiovascular societies and other investigators have also recognized this unmet need and have already published some of their data, but key points are still missing.^6^ Among those are the medium- and long-term outcomes of such patients. For instance, the largest Brazilian CIHD patient registry, the Registry of Clinical Practice in Patients at High Cardiovascular Risk (REACT), has only recently published its one-year outcomes, reporting a 4.92% incidence of death.^6^

Epidemiological studies within the Brazilian population have identified that the rates of adequate control of therapeutic goals and use of prognostic-modifying drugs are both low.^2,6^ However, these studies were performed in the general population or in patients at high cardiovascular risk, but not specifically in patients with coronary artery disease.

Therefore, we sought to improve the characterization of CIHD patients in Brazil. This study aims to report the two-year incidence of death, myocardial infarction (MI), or stroke in Brazilian CIHD patients followed in a tertiary public health care center. As a secondary objective, we attempted to identify prognostic determinants and evaluate medication use and risk factor control in such patients.

## Methods

This is a two-year follow-up report on a prospective observational registry. The registry is currently in use, and this report refers to this specific period. We sought to conform to the STROBE guidelines.^10^

### Setting and patients

The study was conducted at the Instituto do Coração (InCor), São Paulo, Brazil. InCor is a tertiary reference center for high-complexity and high-risk patients with heart disease. These patients have public health insurance and proceed mainly from the state of São Paulo, as well as from other parts of the country. From January 2016 to December 2018, we registered patients with stable CIHD who underwent treatment and care at our outpatient clinic. To be eligible, patients must have had a history of previous coronary artery bypass surgery, percutaneous coronary intervention, or documented coronary artery lesions > 50%. In this analysis, we did not include patients with acute coronary syndromes. However, these patients were eligible for inclusion after discharge if they were to be monitored at the clinic (Central Illustration).

In this investigation, diagnosis of diabetes was based on patients with glycated hemoglobin equal to or greater than 6.5% or those in use of antidiabetic medication. Hypertension was defined as use of any antihypertensive agent.

All patients provided signed consent.

### Measurements and outcomes

Data collection was standardized and patients were evaluated at baseline and each year afterward. Follow-up was done personally whenever possible, or remotely via phone calls.

The primary endpoint was the composite of death, MI, or stroke. There was no event adjudication committee. Instead, we relied on health records and patient reports. Since InCor is a benchmark center for the treatment of CIHD, many of those patients were treated at our facilities. Whenever they were not, they were asked to bring health records from other providers. Death was double-checked on governmental databases. Secondary endpoints comprehended the incidence of death or MI, and the composite of death and MI. All analyses were exploratory in nature.

### Statistical analysis

Normality was assessed by the Shapiro-Wilk test. Continuous data did not present a normal distribution and, therefore, were described using median and interquartile range (25^th^ – 75^th^ percentile). Categorical data is presented as absolute values and percentages. This is an ongoing registry of CIHD patients, with no prespecified study size.

Primary and secondary endpoints are reported as two-year Kaplan-Meier incidences and their respective 95% confidence interval. Hazard ratios (HR) of the prognostic factors were estimated according to the Cox Proportional Hazards modeling. Multivariate modeling was done with a backward stepwise regression algorithm with a p-to-remove of 0.05. All factors with a p-value < 0.10 in the univariate analyses were included in the initial multivariate model. Changes in clinical and laboratorial parameters between evaluations were assessed using McNemar’s and McNemar-Bowker’s tests for categorical variables and using the Wilcoxon signed-rank test for continuous ones.

All analyses were done with R software (version 4.2.2).^11^ A significance level of 0.05 was adopted.

## Results

From January 2016 to December 2018, 625 participants with known CIHD were included in the registry, and the baseline characteristics are described in Table 1. At the median time of 881 days (IQR: 613-1071), 553 patients were reevaluated.

**Table 1:** – Baseline characteristics

	Overall (n=625)
Women, n	207 (33.1%)
Age, years	66.1 [59.6 – 71.9]
BMI, kg/m^2^	27.3 [24.7 – 30.8]
Previous MI, n	391 (62.6%)
Previous CABG, n	202 (32.4%)
Previous PCI, n	296 (47.4%)
Previous PCI or CABG, n	423 (67.7%)
Previous stroke, n	33 (5.3%)
PAD, n	34 (5.4%)
Previous MI, stroke, or revascularization procedure, n	543 (86.9%)
Hypertension, n	517 (83.1%)
Diabetes, n	304 (48.6%)
Chronic kidney failure, n*	205 (34.7%)
Ejection fraction < 40%, n	110 (24.8%)

Continuous variables are described as median [interquartile range]. Chronic kidney failure: creatinine clearance per Cockroft-Gault method < 60mL/min. BMI: body mass index; MI: myocardial infarction; CABG: coronary artery bypass grafting; PCI: percutaneous coronary intervention; PAD: peripheral artery disease.

Table 2 compares the use of medications at baseline and during follow-up. There was no significant difference in the overall prescription of antithrombotic agents (96.6% to 95.3%, p = 0.32). The use of statins decreased (96.6% to 93.7%, p = 0.02). On the other hand, the prescription of angiotensin-converting enzyme inhibitors (ACEi) or angiotensin receptor blockers (ARB) increased (69.4% to 79.7%, p < 0.001). The use of metformin and sulfonylureas also increased (36.7% to 43.9%, p < 0.001; 18.3% to 22.4%, p = 0.01, respectively). There was no difference in the prescription of insulin (11.2% to 13.4%, p = 0.11) or other antidiabetic agents (2.7% to 2.7%, p = 1.00). Considering the combined prescription of medications with known cardiovascular benefit to the CIHD population (ACEi or ARB plus statin plus any antithrombotic agent), a greater proportion of patients were on optimal medical treatment at FU, compared to baseline: 65.8% vs. 73.6%, p < 0.001.

**Table 2:** – Medications at baseline and follow-up

	Baseline (n = 553)	Follow-up (n = 553)	p-value
ASA	516 (93.3%)	491 (88.8%)	< 0.001
Other antiplatelet agent	133 (24.1%)	62 (11.2%)	< 0.001
Oral anticoagulants	28 (5.1%)	34 (6.1%)	0.21
Any antithrombotic agent	534 (96.6%)	527 (95.3%)	0.32
Statin	534 (96.6%)	518 (93.7%)	0.02
Ezetimibe	15 (2.7%)	10 (1.8%)	0.33
Fibrates	31 (5.6%)	25 (4.5%)	0.42
ACEi or ARB	384 (64.9%)	441 (79.7%)	< 0.001
Beta-blocker	492 (89.0%)	468 (84.6%)	< 0.001
Calcium channel blocker	208 (37.6%)	227 (41.0%)	0.09
Nitrates	163 (29.5%)	116 (21.0%)	< 0.001
Ivabradine	2 (0.4%)	3 (0.5%)	1.0
Trimetazidine	5 (0.9%)	6 (1.1%)	1.0
Metformin	203 (36.7%)	243 (43.9%)	< 0.001
Sulfonylureas	101 (18.3%)	124 (22.4%)	0.01
Insulin	62 (11.2%)	74 (13.4%)	0.11
Other antidiabetic agents	15 (2.7%)	15 (2.7%)	1.00
ACEi or ARB + statin + any antithrombotic agent	364 (65.8%)	407 (73.6%)	< 0.001

ASA: acetylsalicylic acid; ACEi: angiotensin-converting enzyme inhibitor; ARB: angiotensin receptor blockers.

At follow-up, patients were less symptomatic (p < 0.001). Systolic and diastolic blood pressure, heart rate, LDL-cholesterol, and HDL-cholesterol were significantly different from baseline measurements (p < 0.05 for all analyses), albeit only mildly in intensity (Table 3). Total cholesterol, triglycerides and glucose were not significantly different from baseline measurements.

**Table 3:** – Clinical findings at baseline and follow-up

	Baseline (n = 553)	Follow-up (n = 553)	Median [25th – 75th percentile] change	p-value
**Angina intensity, n**				< 0.001
No angina	358 (66%)	445 (82%)		
CCS 1	71 (13%)	39 (7%)		
CCS 2	93 (17%)	45 (8%)		
CCS 3 or 4	23 (4%)	16 (3%)		
Systolic blood pressure, mmHg	130 [120 – 140]	130 [120 – 150]	0 [-10 – 20]	< 0.01
Diastolic blood pressure, mmHg	80 [70 – 80]	80 [70 – 90]	0 [-10 – 10]	0.03
Heart rate, bpm	64 [60 – 72]	68 [61 – 72]	0 [-5 – 8]	0.02
Serum cholesterol, mg/dL	152 [130 – 184]	155 [131 – 188]	1 [-16 – 22]	0.054
LDL-cholesterol, mg/dL	83 [64 – 108]	83.5 [64 – 112]	1.5 [-13 – 18]	0.03
HDL-cholesterol, mg/dL	43 [36 – 51]	41 [36 – 49.2]	-1 [-5 – 3]	0.01
Triglycerides, mg/dL	119 [82 – 159]	118 [85 – 168]	-1 [-26.5 – 32.5]	0.64
Glucose, mg/dL	115 [103 – 143]	115 [103 – 154]	0 [-11 – 13.5]	0.32

Continuous variables are described as median [interquartile range]. CCS: Canadian Cardiovascular Society; LDL-cholesterol: low-density lipoprotein cholesterol; HDL-cholesterol: high-density lipoprotein cholesterol.

Compared to baseline evaluation, more patients were asymptomatic (defined as CCS 0; 65.2% vs 81.7%, p < 0.01) and fewer met the SBP < 140 mmHg therapeutic goal (67.8% vs 61.0%, p = 0.01) at follow up (Figure 1). Roughly a third of patients met the LDL-c < 70 mg/dL therapeutic goal both at baseline and follow-up (p = 0.74). Out of the 358 asymptomatic patients at baseline, 47 (13.1%) developed angina during follow-up (Figure 2). Conversely, 54/71 (76.1%), 65/93 (69.9%), and 15/23 (65.2%) of those with angina grades 1, 2, or 3/4, respectively, became asymptomatic.

**Figure 1:** – Number of patients that achieved clinical goals. ACEi: angiotensin-converting enzyme inhibitor; ARB: angiotensin receptor blockers; Antithrombotic: antiplatelets and/or oral anticoagulants; LDL-c: low-density lipoprotein cholesterol; SBP: systolic blood pressure.

**Figure 2:** – Angina intensity at baseline and follow-up. CCS: Canadian Cardiovascular Society; Asymptom.: asymptomatic.

After a median follow-up of 881 days, 37 events were recorded for the primary endpoint (Table 4). The two-year incidence of death, MI, or stroke was 7.05%. On unadjusted analysis (Table 5), age (HR 1.58 per five years, 95% CI 1.35 - 1.85), previous stroke (HR 3.11, 95% CI 1.38 - 7.00), LDL-cholesterol (HR 1.20 per 10 mg/dL increase, 95% CI 1.11 - 1.30), and total cholesterol (HR 1.14 per 10 mg/dL, 95% CI 1.07 - 1.22) were associated with an increase in the primary endpoint. After adjustments, age (HR 1.61 per five-year increase, 95% CI 1.32 - 1.97), previous stroke (HR 3.65, 95% CI 1.48 - 9.00), and LDL-cholesterol (HR 1.23 per 10 mg/dL, 95% CI 1.14 - 1.33) were independently associated with the primary endpoint (Figure 3).

**Table 4:** – Two-year incidence of endpoints

	Number of events	2-year incidence (95% CI)
Death, MI, or stroke	37	7.05% (4.81 - 9.23)
Death or MI	35	6.67% (4.49 - 8.80)
Death	31	5.87% (3.83 - 7.87)

MI: myocardial infarction; CI: confidence interval.

**Table 5:** – Unadjusted and adjusted Cox Proportional Hazards models for incidence of myocardial infarction, stroke, or death

	Unadjusted		Adjusted*	
	Hazard Ratio (95% CI)	p-value	Hazard Ratio (95% CI)	p-value
Women	0.97 (0.52; 1.80)	0.93		
Age, per 5 years	1.58 (1.35; 1.85)	< 0.005	1.61 (1.32; 1.97)	< 0.001
BMI, per 5 kg/m^2^	0.83 (0.60; 1.16)	0.28		
Previous MI	1.40 (0.74; 2.67)	0.30		
Previous CABG	1.79 (1.00; 3.20)	0.05		
Previous PCI	0.72 (0.40; 1.30)	0.28		
Previous Stroke	3.11 (1.38; 7.00)	0.01	3.65 (1.48; 9.00)	< 0.001
PAD	0.30 (0.04; 2.17)	0.23		
Diabetes	0.88 (0.49; 1.58)	0.67		
Ejection fraction, per 5%	0.90 (0.79; 1.02)	0.10		
Angina	1.04 (0.57; 1.89)	0.90		
Hypertension	1.50 (0.59; 3.81)	0.39		
HR, per 10 bpm	0.79 (0.55; 1.15)	0.22		
LDL-c, per 10 mg/dL	1.20 (1.11; 1.30)	< 0.001	1.23 (1.14; 1.33)	< 0.001
HDL-c, per 10 mg/dL	1.06 (0.80; 1.41)	0.69		
Cholesterol, per 10 mg/dL	1.14 (1.07; 1.22)	< 0.001		
Triglyceride, per 10 mg/dL	0.98 (0.94; 1.03)	0.41		

Adjustments were made pursuant to the backward stepwise algorithm with p-to-enter < 0.10 and p-to-remove 0.05, according to univariate results. MI: myocardial infarction; CABG: coronary artery bypass graft surgery; PCI: percutaneous coronary intervention; PAD: peripheral artery disease; HR: heart rate; BMI: body mass index; LDL-c: low-density lipoprotein cholesterol; HDL-c: high-density lipoprotein cholesterol.

**Figure 3:** – Incidence of myocardial infarction, stroke, or death, stratified by independent prognostic factors. LDL: low-density lipoprotein.

## Discussion

To our knowledge, this is one of the largest CIHD registries carried out in Brazil involving stable patients. Overall, we found a two-year incidence of death, MI, or stroke of 7.05%. Age, previous stroke, and high LDL-cholesterol were the main associated risk factors.

Despite efforts that may suggest otherwise, international registries may over- or underrepresent countries and regions.^4,12^ Therefore, reports like this are valuable to better understand the burden of CIHD both regionally and worldwide. In this study, we found that, despite a high percentage of statin prescriptions, less than a third of the patients had LDL-cholesterol lower than 70 mg/dL, and less than 10% had levels lower than 50 mg/dL.

The *Estudo Longitudinal da Saúde do Adulto* (ELSA) study, a Brazilian multi-regional general-population registry, reported that 9.4% of high-risk coronary heart disease participants had LDL-cholesterol lower than 70 mg/dL.^8^ The REACT registry, another multicentric Brazilian registry of high-risk and manifest atherosclerotic disease patients, also reported that > 90% of its participants in secondary prevention had LDL-cholesterol > 50 mg/dL.^6^ This data shows that most patients do not meet the LDL-cholesterol goal of 50-55 mg/dL defined by the guidelines.^13-15^ When considering non-HDL-cholesterol, even fewer (16.7%) patients met the national goal of 80 mg/dL proposed by the guidelines.^13^

This is alarming, as our and previous studies^16,17^ have shown that higher LDL-cholesterol levels are associated with cardiovascular events. Previous studies estimated that each 1 mmol/L (roughly 38 mg/dL) reduction in LDL-cholesterol leads to a 22% reduction in major cardiovascular events over five years.^16^ As a comparison, we estimated a 20% increase in risk per 10 mg/dL – a higher effect estimate. Furthermore, we identified that very few patients were prescribed ezetimibe (< 5%) and none of the patients were using PCSK-9 inhibitors. Ezetimibe has been proposed as a 2^nd^ line drug for those who have not reached adequate LDL-cholesterol levels.^13,15^ We attribute such a scenario to the fact that these medications are not provided by the public health care system, on which most Brazilians (and our participants) rely. We believe that including them in the public health care system would be an effective way of improving the quality of care for CIHD patients.

In our study, LDL and total cholesterol control during follow-up proved to be poor (p < 0.05). Despite this, its impact was small from a clinical point of view (4.1mg/dL increase in LDL and 5mg/dL increase in total cholesterol). The same can be seen in systolic blood pressure (a 3 mmHg increase at follow-up). This decline in the control of comorbidities occurred despite a high statin prescription rate (above 90% at baseline and at follow-up) and an increase in the prescription of ACE inhibitors or ARBs (p < 0.001). There was also an increase in the prescription of oral antidiabetics, which did not improve glycemic control. These data can be markers of poor adherence to treatment.

It could also be the case that LDL-cholesterol decrease is a marker of better patient adherence. Many studies have already demonstrated that patient adherence is an important prognostic factor, and it has even been called “the next frontier in quality improvement.”^18-20^ Previous reports estimate that non-adherence increases major cardiovascular events by about 18%.^20^ Although we have not formally evaluated it, one could interpret low LDL-cholesterol as a proxy of statin adherence (and perhaps the treatment as a whole). Indeed, previous inquiries showed that as much as 30% of Brazilians with chronic non-communicable diseases may be non-adherent, and that Latin Americans may be even more non-adherent than their North American counterparts.^21,22^ As non-adherence could be related to health system- and provider-related issues, further investigations may have an impact on public health policies. Interestingly, there was an improvement in the control of angina. The number of patients without angina increased (p < 0.001) and all other functional classes tended to decrease.

The ProspeCtive observational LongitudinAl RegIstry oF patients with stable coronary arterY disease (CLARIFY) registry, a contemporary international CIHD registry, reported a five-year incidence of cardiovascular death, MI, or stroke of 9.5%.^23^ The REduction of Atherothrombosis for Continued Health (REACH) registry, a larger but slightly older international registry, reported one- and three-year event rates of vascular death, MI, or stroke of 4.5% and 11.6%, respectively, for the CIHD subgroup.^24^ The REACT registry reported a one-year mortality rate of 4.9%. Direct comparisons between studies are difficult to interpret due to differences in inclusion criteria, population, timeframe, and analyzed risk factors.

Yet, our results seem to be in line with international registries, and slightly better than the Brazilian one. Nonetheless, all studies reported that outcomes vary significantly according to geographical region, which further underscores the importance of regional studies and adequate representation in international ones to have a better grasp of the burden of atherosclerosis worldwide.^6,23,24^

Polyvascular disease has been shown to be an important prognostic risk factor, both in chronic and acute coronary syndrome patients worldwide.^12,25^ So has a previous ischemic event, with an associated 40-50% risk increase in further major cardiovascular events.^6,12,23^ Coincidentally, previous stroke was an independent risk factor in our registry. Previous stroke in our population denotes polyvascular atherosclerotic disease (as all patients had CIHD per inclusion criteria). In our population, previous stroke was the most strongly associated risk factor, representing an estimated almost four-fold increase in event incidence. Nonetheless, previous stroke and peripheral (that is, lower limb) artery disease had very low prevalence in our cohort: 5.3% and 5.4%, respectively. These values are lower than those reported in other studies, and could indicate a degree of underdiagnosis.^6,12,26^

Typically, reduction in left ventricular ejection fraction, previous MI, and chronic kidney disease are markers of poor prognosis in patients with CIHD.^23,27^ In our study, we did not observe this prognostic correlation. This absence of correlation could be explained, in part, by the small number of patients with these comorbidities. Another factor that could contribute to this result is the median follow-up of two years and the low number of events in our population.

Our study has several limitations. First of all, we only included patients from one specialized center, so we cannot claim to be a representative sample from CIHD in Brazil as a whole. Second, although this is one of the largest CIHD registries in the country, it still is relatively small in comparison to other international registries. The low number of events (n=37) also precludes more accurate subgroup or risk factor analyses. Third, we did not have a formal event adjudication committee – we relied on patient reports, health records, and administrative data whenever possible. Hence, some events may have gone undetected. Fourth, all analyses were exploratory and there is a high probability of type I error; therefore, care should be taken while interpreting them.

## Conclusion

In conclusion, we have shown that CIHD patients in our center presented a two-year incidence of death, stroke, or MI of 7.05%, which is in accordance with major international registries. In this setting, we also identified LDL-cholesterol as the main modifiable risk factor and possible valuable target for further improvement in health care.
